# Diving into the Digital Landscape: Assessing the Quality of Online Information on Neonatal Jaundice for Parents

**DOI:** 10.3390/children11070877

**Published:** 2024-07-19

**Authors:** Michael Karl Baumgartner, Anna-Lena Behr, Anne Christina Garbe, Christoph Quatember, Heiko Reutter, Joachim Woelfle, Fabian Benedikt Fahlbusch, Gregor Hanslik

**Affiliations:** 1 Division of Neonatology and Pediatric Intensive Care Medicine, Department of Pediatrics and Adolescent Medicine, Friedrich-Alexander-University of Erlangen-Nürnberg, 91054 Erlangen, Germany; michael.baumgartner@uk-augsburg.de (M.K.B.); gregor.hanslik@uk-erlangen.de (G.H.); 2 Neonatology and Pediatric Intensive Care, Faculty of Medicine, University of Augsburg, 86156 Augsburg, Germany; 3 Department of Pediatrics and Adolescent Medicine, Friedrich-Alexander-University of Erlangen-Nürnberg, 91054 Erlangen, Germany

**Keywords:** jaundice, online information, neonatal care

## Abstract

Background: Hyperbilirubinemia is a common condition in newborns. While mild cases of jaundice are common and typically resolve spontaneously, severe hyperbilirubinemia can lead to serious neurologic complications if left untreated. With the constant adaptation of guidelines, clinical management has significantly improved, and treatment has become routine for pediatricians. However, for parents of affected children, managing the condition is not routine. In today’s digital age, parents often seek additional information by accessing a wide range of medical resources on the internet. While this can be empowering, it also presents challenges, as the quality and accuracy of online medical information can vary widely. Therefore, we analyzed the current quality of information on jaundice found on the internet by parents. Methods: A simulated internet search (using the Google search engine) was conducted from a layperson’s perspective using German (“Neugeborenes Gelbsucht”, “Baby Gelbsucht”) and English (“jaundice newborn”, “jaundice baby”) search terms. Subsequently, the quality of the search results was assessed by two independent neonatologists based on the DISCERN Plus Score, HONcode certification, and the JAMA criteria. Results: Websites targeting non-medical laypersons exhibited significant variability in quality. Notably, the content of English websites was superior to that of websites in the German language. The majority of English sites were predominantly institutional, whereas most German sites were commercially oriented. Conclusions: Although information on jaundice is readily accessible online for non-medical individuals, there were notable differences in quality based on language and significant variability in the quality of information warranting attention from healthcare professionals. Furthermore, German websites providing information on jaundice were often hosted by commercial organizations. We propose that pediatric societies engage in developing and maintaining organization-based medical information to improve online resources for parents.

## 1. Introduction

The management of jaundice remains a global challenge [1]. It commonly occurs in neonates following postnatal discharge and is mostly observed by parents as a yellowing of the skin, increased sleepiness, and a reduction in feeding behavior [2]. While this typically represents a benign physiological condition for most newborns, approximately one in ten develop clinically significant hyperbilirubinemia, necessitating the prompt administration of an appropriate inpatient treatment for the prevention of bilirubin encephalopathy [3,4]. Groundbreaking advancements in perinatology, such as the research carried out on Rhesus prophylaxis in the 1960s by Finn et al. [5] and Freda et al. [6] and the research carried out on phototherapy in 1958/1960 by Cremer et al. [7] and Ferreira et al. [8], as well as the recent improvement in jaundice monitoring via transcutaneous bilirubin detection methods in addition to total serum bilirubin (TSB) levels [9,10], have greatly diminished the fear of this complication. Interestingly, however, the incidence of bilirubin encephalopathy remains significant, even in high-income countries (HICs) [11,12]. The failure to achieve the millennium goal of making kernicterus a never event underscores the ongoing challenge in healthcare systems [13]. While the number of affected newborns in HICs is relatively low, the neurological consequences and associated impairments of bilirubin encephalopathy impose significant burdens on affected children and their families [14]. In addition to the early detection of severe hyperbilirubinemia in newborns and prompt initiation of therapy, educating parents comprehensively is crucial to further reducing or preventing encephalopathy.

In today’s digital age, parents increasingly turn to the internet for health-related information [15,16,17], including guidance on managing their child’s health concerns, such as jaundice. The accessibility and abundance of online resources make it a convenient source of information for many, shaping their understanding and decisions regarding healthcare. The British Ofcom Online Nation Report has shown that health-related internet usage among adults was at 67% (including parents) in 2023, and among children (8–15 years), it was at 16% [18].

However, the quality and reliability of online health information vary widely, posing challenges for both parents and healthcare providers in ensuring accurate and informed decision-making. It is noteworthy that even back in 2002, 22% of parents consulted the internet before visiting their healthcare provider with their child. However, only 34% of these parents shared this information with their physician [17].

Such findings underscore the necessity to understand the patterns of online health information seeking among parents and the quality of content for sufficient healthcare delivery today. This knowledge helps to inform strategies to improve the dissemination of accurate information, promote health literacy, and enhance the partnership between parents and healthcare professionals in managing conditions such as neonatal jaundice effectively [19,20]. Against this backdrop, our investigation into the online health information landscape for neonatal jaundice revealed a gap in research on the quality of information available to parents about this condition. Therefore, our objective was to conduct a standardized evaluation of the current quality of online German- and English-language information resources for parents.

## 2. Methods

Quality assessment of websites using HONcode, JAMA score, and modified DISCERN Plus Score (DPS) has been previously described by us in detail [21,22,23]. In short, identification and selection of websites were performed on 11 May 2023 (9–10 p.m. CET) using Google as a search engine. The search engine Google was selected due to its popularity and frequent use by patients [24]. The decision to limit the search to the top 20 results was based on studies indicating that individuals typically restrict their web access to the top search results [25]. In fact, the majority of users access only the top 5 websites [24]. German and English language websites were evaluated separately due to documented discrepancies in quality [22,26]. This approach also highlights potential differences in the quality of medical information across different healthcare systems and regulatory environments of HICs. Understanding the quality of information in these two languages might highlight areas with potential for improvement in public health communication. Moreover, English is considered the lingua franca of medical research and is the predominant language in scientific publications.

Additionally, English is the predominant language of the internet, transcending generational demographics [27]. Therefore, we analyzed both German and English online resources on hyperbilirubinemia to assess the scope and quality of information available on both national and international scales. The following English and German keywords were entered into the search engines Google.de and Google.com: “Neugeborenes/Baby Gelbsucht” and “Newborn/Baby Jaundice”. This helped to emulate real user experience. Before conducting the search, the PC location was set to Germany (Berlin) and the USA (Washington D.C.) using a virtual private network (VPN). The browser cache was cleared, and cookies and search histories were deleted. For the respective searches, the language filter and result region were set to German/Germany and English/United States. Exclusion criteria included the following: limited information on the search topic (less than one paragraph), restricted access requiring a password, repeated server unavailability, and direct forwarding to other domains. Based on their hosting, websites were categorized into the following groups: medical news sites, international governmental (IG) and non-governmental organizations (NGOs), educational sites, governmental sites, and medical center/university-owned sites.

The top 20 search results were downloaded for offline evaluation by two independent board-certified neonatologists (MKB and GH) using a standardized evaluation chart, provided in Supplementary File S1. There was some overlap in the results for the two keywords, resulting in 25 German sites and 24 English sites for the final analysis. Interrater discrepancies were discussed with a third party (FBF) to reach a consensus. The proportion of agreement beyond chance was substantial, with a Cohen’s kappa greater than 0.61 (see below [28]).

### 2.1. Modified DISCERN Plus Score [23,29,30]

The DISCERN instrument is a validated tool for assessing the quality of written medical information based on 15 questions, with scores ranging from 1 (definitely no) to 5 (definitely yes). The tool evaluates reliability and treatment details across two sections. We adapted the second section to better fit our study context (see Supplementary File S1), including a 16th question on overall quality, and rated websites as “excellent”, “good”, “fair”, “poor”, or “very poor” based on their final scores (maximum 80), as described by Nghiem et al. [31].

### 2.2. HONcode

The Health On the Net Foundation’s Code of Conduct (HONcode, www.healthonnet.org) is a certification system ensuring quality, objective, and transparent medical information. We recorded whether each website was HONcode-accredited by using the respective browser plug-ins. Certification indicates compliance with standards such as author credentials, document modification dates, data confidentiality, data sources, funding, and advertising policies [32].

### 2.3. JAMA Benchmarks

The Journal of the American Medical Association (JAMA) benchmarks provide website guidelines for authorship, attribution, disclosure, and accuracy in relation to medical information [33]. We assessed each website’s adherence to these benchmarks to indirectly evaluate webpage quality.

### 2.4. Statistical Analysis

Statistical analysis was performed using GraphPad Prism 10.2.3 (GraphPad Software Inc., La Jolla, CA, USA). The following tests were employed: Spearman correlation analysis, linear regression analysis, and a two-tailed non-parametric Wilcoxon–Mann–Whitney test for group comparisons. A *p*-value of <0.05 was deemed statistically significant. For the Modified DISCERN Plus Score, Cohen’s κ [28] was applied to assess interobserver variability/interrater reliability via a quadratic Cohen’s weighted kappa test after correction for agreement by chance using an online tool [34]. This test took into account the degree of matching between the ratings, as well as the extent of their discrepancies. Kappa values of κ = 0.61–0.80 were interpreted to show substantial agreement [28,35,36]. For the overall modified DPS, Cohen’s weighted κ (quadratic) yielded a κ value of 0.709 (German) and a κ value of 0.731 (English) as the proportion of agreement beyond chance (z = 3.61, *p* = 0.0003 and *p* = 0.00031, respectively).

## 3. Results

### 3.1. Website Hosting

The internet search yielded *n* = 25 and *n* = 24 German and English homepages, respectively. Of the jaundice-related websites in German, the majority (*n* = 14) were sponsored medical news sites, 35.7% of which were among the top 10 Google hits. Overall, 5 homepages were hosted by interest groups/non-governmental organizations (IG/NGO) and educational organizations (EDU, *n* = 1 in the top 10), respectively. A single homepage was established by the German government. One search hit was Austrian.

The jaundice-related websites in English were hosted in the United Kingdom (*n* = 1), Canada (*n* = 2), Australia (*n* = 3), and USA (*n* = 18). Nine homepages were established by medical centers/universities and the government, respectively (four among the top ten, each). In contrast to German search hits, medical news sites (*n* = 1) were barely represented among the top 20 Google search results with EDU (*n* = 2) and IG/NGO (*n* = 3).

### 3.2. Quality Assessment

**Figure 1:**
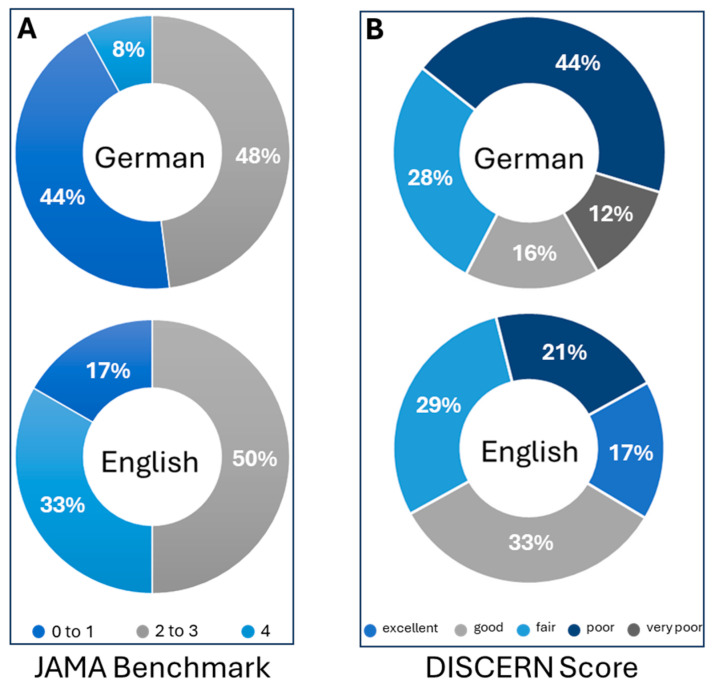
Sunburst diagrams displaying staggered JAMA benchmarks (**A**) and DISCERN Plus Score results (**B**). The assessment tool analyzes the clarity, balance, and content of the information found on websites and allows for grading from 1 (poor) to 5 (excellent). The first eight questions address reliability; the next seven questions focus on treatment information. DISCERN Plus minimum score = 16; maximum score = 75. Question 16 rates the overall quality, and it was not included in the total DISCERN Plus Score. JAMA benchmark criteria: minimum score = 0; maximum score = 4.

**Figure 2:**
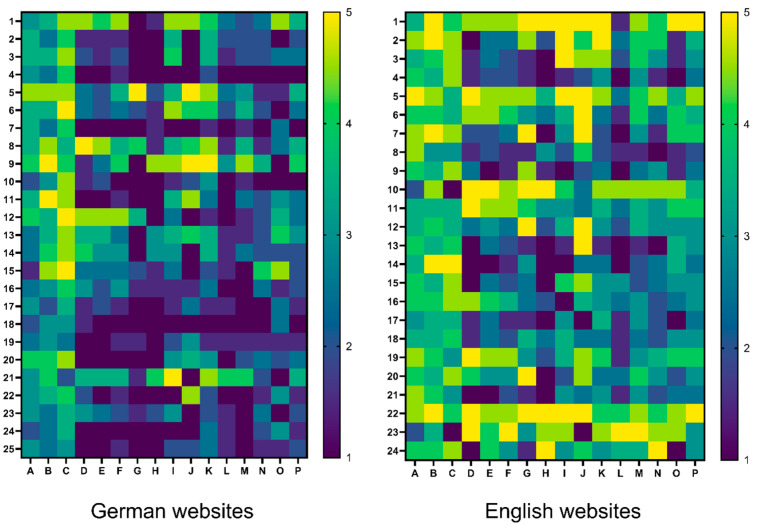
Heatmap analysis of the mean DISCERN Plus Score in German and English websites on jaundice. Legend: the heatmap shows the average DISCERN Plus Scores for German (**left**) and English (**right**) websites on jaundice. *Y*-axis: the numbers (1 to 25 for German and 1 to 24 for English) represent the top search results on Google. *X*-axis: the letters (A–P) correspond to different DISCERN questions: A–H: questions about the reliability of the information. I–O: questions about the quality of treatment information provided. P: the overall rating of the website. The colors indicate the scores for each item: brighter colors represent higher DISCERN scores, while dark colors represent lower DISCERN Plus Scores, with blue representing the lowest score (1) and yellow representing the highest score (5).

JAMA benchmark (Figure 1A) and modified DISCERN Plus Score (Figure 1B) analyses of the German and English websites revealed a lack of very poor sites using English search terms. However, 56% of the German websites on jaundice were of very poor to poor quality (Figure 1A). German search terms did not return homepages with DISCERN Plus Scores of >63 (Figure 1B). In contrast, using English search terms resulted in 17% of the websites being excellent websites (Figure 1B). The heatmap analysis (Figure 2) gives a detailed overview of the mean DISCERN Plus Score per website and item aiding the visualization of quality. In general, English websites showed more heat unrelated to certain items or categories.

A comparison of DISCERN scoring results regarding reliability and treatment information between the English and German websites (Table 1) indicated a higher quality in both these categories when English search terms were entered into Google.

**Figure 3:**
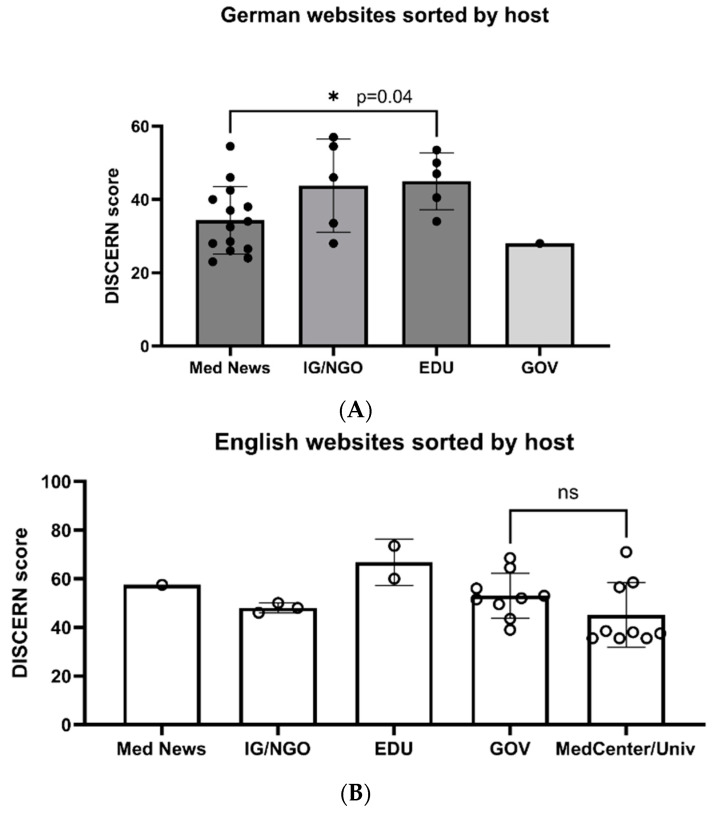
German (**A**) and English (**B**) websites on jaundice sorted by host. Legend: Med News = medical news site (sponsored), IG/NGO = interest groups/non-governmental organizations, EDU = educational organizations, GOV = governmental organizations, and MedCenter/Univ = medical center/university. Bar color: gray—German websites (**A**); white—English websites (**B**). ns = *p* > 0.05, * indicates statistical significance.

Figure 3A,B show differences in the DISCERN Plus Score regarding the hosting source. A detailed heatmap analysis is given in Supplementary Figure S1. The German sponsored medical news sites (Figure 3A) had a significantly lower score (34.32 ± 9.22; mean ± SD) when compared to the EDU sites (45.0 ± 7.79; *p* = 0.04). The quality of the German IG/NGO sites was comparable to the analyzed EDU sites; however, this result did not reach significant difference, based on its standard deviation (43.8 ± 12.74). English homepages (Figure 3B) were predominately of governmental origin or hosted by medical centers/universities. There was no significant difference in DISCERN Plus Scores between these categories (53.06 ± 9.24 vs. 45.17 ± 13.27, respectively).

### 3.3. Difference between Quality Ranking and Google Ranking

**Figure 4:**
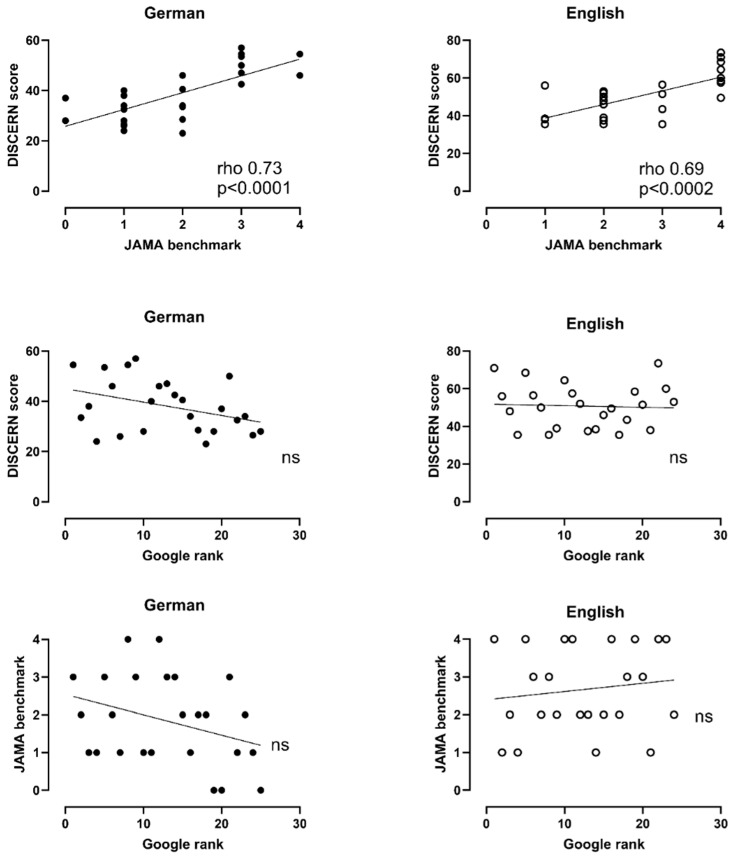
Spearman correlation analysis of DISCERN Plus Score, JAMA benchmark and Google rank. DISCERN Plus Score correlates positively with the JAMA benchmark. Results from both assessment tools did not correlate with the respective Google ranks.

Using Spearman correlation analysis (Figure 4), we found a positive correlation between the DISCERN Plus Score and the JAMA benchmark for both the German-language (*p* < 0.0001) and the English-language websites (*p* < 0.0002). There was no correlation between DISCERN Plus Score and the JAMA benchmark with the respective Google ranks.

### 3.4. HONcode Certification

Overall, 20% of jaundice-related websites in German were HONcode-certified (EDU *n* = 2, IG/NGO *n* = 1, medical news site (sponsored) *n* = 2), with only 2 certified sites among the top 10 Google search results. In contrast 58% of the respective English homepages were certified, with 2 uncertified sites among the top 10 Google search hits. Among both the German and English websites, no significant quality difference in DISCERN Plus Score and JAMA benchmark was observed between the websites certified by the HONcode and those that were not.

## 4. Discussion

The use of standardized tools enabled us to determine the quality of online information on jaundice in neonates. Aiming to uncover areas with potential for improvement, our analysis revealed significant variability in the quality of websites targeting non-medical laypersons. Notably, English-language websites generally offered superior content compared to their German-language counterparts. Furthermore, the majority of English websites were predominantly institutional, while most German websites were commercially oriented.

Our analysis raises the question of whether the current quality of information about neonatal jaundice on the web reflects the level of awareness among parents. Early recognition of neonatal hyperbilirubinemia is essential for preventing bilirubin neurotoxicity and its long-term sequelae. A global meta-analysis conducted by Farouk et al. in 2020 [37] showed a pooled estimate of societal awareness of neonatal hyperbilirubinemia of 67%, highlighting a significant need to improve awareness. This study focused on low- and middle-income countries (LMICs) and identified hospital location as a crucial determinant of awareness of complications. While it might be concluded that awareness is low, the estimated incidence of bilirubin encephalopathy in HICs is 1 in 67,000 neonates [38,39], which supports the argument for comprehensive neonatal care in these regions. Therefore, caregivers should recognize their responsibility to provide preventive medical education, especially in Germany, which is currently undergoing a transformation to centralized neonatal medical care [40].

Interestingly, in Germany, this high standard of care was not reflected in the top search ranks for educational, governmental, and university-based content. Our finding that the DISCERN Plus Score and JAMA ranking differed significantly from the Google search rank suggests that the search engine’s ranking algorithm did not necessarily prioritize the quality or reliability of health information, in contrast to its self-imposed goal. Several factors could have contributed to this discrepancy, such as Search Engine Optimization (SEO) practices and so-called engagement metrics [41]. For the former, websites that invest heavily in SEO can achieve higher rankings regardless of the quality of their content. Thus, commercially driven websites with effective SEO strategies might rank higher than more reliable, evidence-based resources, which might have been the case for our German-language based results. Commercial websites often have more resources to invest in SEO and online marketing, allowing them to achieve higher search ranks. These websites may prioritize attracting visitors over providing high-quality, research-based information. In this regard, Google’s algorithm often considers user engagement metrics such as click-through rate and time spent on the page, which do not necessarily correlate with the accuracy or reliability of the information. Popular websites or those that generate more user interaction may rank higher despite offering lower-quality content. Additionally, websites that frequently update their content or have a large volume of information might be favored by search engines. However, frequent updates or extensive content libraries do not always equate to reliable information of high quality. In summary, search algorithms are not specifically designed to assess the medical accuracy or reliability of information and do not always align with the principles of high-quality health communication.

Given these findings, it is evident that there is a critical need for improvement in the visibility of high-quality medical information in search engine results. Public health organizations and medical societies should consider strategies to enhance the online presence of reliable, evidence-based health information to ensure that laypersons have access to trustworthy resources when searching for medical information online.

Moreover, empowering parents through medical education is crucial for establishing cost-effective outpatient treatment options in neonatology, such as transcutaneous bilirubin monitoring or home phototherapy [42,43].

### Limitations and Future Aspects

We did not investigate the prevalence of web scraping, content duplication, the proliferation of low-quality or spam websites for commercial purposes, and SEO spamming in our study. Recently, Bevendorff et al. [44] discussed the impact of such practices on search engine rankings, user experience, and the reliability of online information in detail. The findings and discussion raise the concern that such practices might ultimately lead to a further decrease in the quality of information found on the internet. Website cloning for commercial purposes could potentially dominate search engine results, negatively affecting users’ access to reliable information.

Moreover, as previously discussed, the reading level or educational level of the parents significantly influences their understanding of web content [23]. While our study did not account for these confounders, the Health Literacy Surveys Germany 1 and 2, conducted in 2017 and 2021 [45,46], indicate that the health literacy of the German population is generally low. In 2021, 58.8% of participants had limited health literacy. Many respondents reported difficulties in accessing (48.3%), understanding (47.7%), and applying (53.5%) information, with an even higher percentage (74.7%) experiencing difficulties in appraising information [46]. These findings undermine the improving English proficiency in Germany [47].

The layout of our study design led to the retrieval of only websites from HICs. Germany, for instance, has a population with a migration background comprising 20 million people, with these people predominantly being from LMICs [48]. Within this demographic, approximately 60% rely on the internet as their primary source of health information, and over 70% utilize health-related websites. However, as demonstrated by Schaeffer et al., this group exhibits a low level of health literacy [49]. This highlights a potential gap in accessible, high-quality online health information for populations with a migration background in countries like Germany. Given that a significant portion of this demographic relies on the internet for health information yet exhibits low health literacy, there is a critical need for tailored public health interventions to enhance the quality and accessibility of online medical information for these vulnerable groups.

## 5. Conclusions

Due to the poor quality of sites composed in German, it remains crucial for midwives and pediatricians to carefully and conscientiously inform and educate parents about neonatal jaundice, the risk of bilirubin encephalopathy, and the importance of monitoring and appropriate therapy. Given the high quality of English-language websites created by medical centers, universities, and state institutions, it raises the question of whether there should be a demand for similar high-quality medical information websites in Germany. We propose that pediatric societies engage in developing and maintaining organization-based medical information to improve online resources for parents.

## Figures and Tables

**Table 1:** Descriptive statistical analysis of DISCERN Plus Score results. Score description is given in Figure 1. Statistical analysis was performed using a non-parametric Wilcoxon-Mann–Whitney test.

Descriptive Statistics (Mean ± SD)			
DISCERN Score	German Websites (*n* = 25)	English Websites (*n* = 24)	*p*-Value
**Reliability**	20.24 ± 5.47	26.15 ± 5.80	*p* = 0.0007
*Q1-8*			
**Treatment Information**	15.64 ± 5.08	21.38 ± 5.72	*p* = 0.0005
*Q9-15*			
**Total**	38.1 ± 10.61	50.79 ± 11.61	*p* = 0.0004
*Q1-15*			
**Overall Quality**	2.22 ± 0.89	3.27 ± 0.79	*p* = 0.0001
*Q16*			
**JAMA Benchmark**	1.84 ± 1.18	2.67 ± 1.13	*p* = 0.021

## Data Availability

All necessary data are included within the manuscript. Additional data supporting the findings of this study are available from the corresponding author, F.B.F., upon reasonable request.

## Supplementary Materials

The following supporting information can be downloaded at: https://www.mdpi.com/article/10.3390/children11070877/s1, Supplementary File S1: Standardized chart for the quality assessment of websites, Supplementary Figure S1: Heatmap analysis of the mean DISCERN Score in German and English websites on jaundice regarding the hosting source.

## References

[1] Olusanya B.O., Kaplan M., Hansen T.W.R. (2018). Neonatal hyperbilirubinaemia: A global perspective. Lancet Child Adolesc. Health.

[2] Bhutani V.K., Stark A.R., Lazzeroni L.C., Poland R., Gourley G.R., Kazmierczak S., Meloy L., Burgos A.E., Hall J.Y., Stevenson D.K. (2013). Predischarge Screening for Severe Neonatal Hyperbilirubinemia Identifies Infants Who Need Phototherapy. J. Pediatr..

[3] Schneider A.P. (1986). Breast Milk Jaundice in the Newborn: A Real Entity. JAMA.

[4] National Collaborating Centre for Women’s and Children’s Health (UK) (2010). Neonatal Jaundice.

[5] Finn R., Clarke C.A., Donohoe W.T., McConnell R.B., Sheppard P.M., Lehane D., Kulke W. (1961). Experimental studies on the prevention of Rh haemolytic disease. Br. Med. J..

[6] Freda V.J., Gorman J.G., Pollack W. (1964). Successful Prevention of Experimental Rh Sensitization in Man with an Anti-Rh Gamma_2_-Globulin Antibody Preparation: A Preliminary Report. Transfusion.

[7] Cremer R.J., Perryman P.W., Richards D.H. (1958). Influence of Light on the Hyperbilirubinæmia of Infants. Lancet.

[8] Ferreira H.C., Cardim W.H., Mellone O. (1960). Phototherapy. A new therapeutic method in hyperbilirubinemia of the newborn. J. Pediatr. Rio J..

[9] Hannemann R.E., Dewitt D.P., Wiechel J.F. (1978). Neonatal Serum Bilirubin from Skin Reflectance. Pediatr. Res..

[10] Yamanouchi I., Yamauchi Y., Igarashi I. (1980). Transcutaneous Bilirubinometry: Preliminary Studies of Noninvasive Transcutaneous Bilirubin Meter in the Okayama National Hospital. Pediatrics.

[11] Alkén J., Håkansson S., Ekéus C., Gustafson P., Norman M. (2019). Rates of Extreme Neonatal Hyperbilirubinemia and Kernicterus in Children and Adherence to National Guidelines for Screening, Diagnosis, and Treatment in Sweden. JAMA Netw. Open.

[12] Arenz S., Franke I., Giani G., Griese M., Grote V., Hahn A., von Kries R., Lainka E., Schaaff F., Schmitt H.J. (2024). ESPED-Jahresbericht 2005. https://www.unimedizin-mainz.de/typo3temp/secure_downloads/43045/0/b5b155c936d4c1b471b7490e1bf1b3af2e301bd1/Jahresbericht_2005.pdf.

[13] Davidson L., Thilo E.H. (2003). How to Make Kernicterus a “Never Event”. NeoReviews.

[14] Shapiro S., Le Pichon J.B., Riordan S.M., Watchkoe J. (2017). The Neurological Sequelae of Neonatal Hyperbilirubinemia: Definitions, Diagnosis and Treatment of the Kernicterus Spectrum Disorders (KSDs). Curr. Pediatr. Rev..

[15] Wainstein B.K., Sterling-Levis K., Baker S.A., Taitz J., Brydon M. (2006). Use of the Internet by parents of paediatric patients. J. Paediatr. Child Health.

[16] Khoo K., Bolt P., Babl F.E., Jury S., Goldman R.D. (2008). Health information seeking by parents in the Internet age. J. Paediatr. Child Health.

[17] Tuffrey C. (2002). Use of the internet by parents of paediatric outpatients. Arch. Dis. Child..

[18] Ofcome.org.uk 2023 Ofcom Online Nation 2023 Report. https://www.ofcom.org.uk/__data/assets/pdf_file/0029/272288/online-nation-2023-report.pdf.

[19] Zaidman E.A., Scott K.M., Hahn D., Bennett P., Caldwell P.H. (2023). Impact of parental health literacy on the health outcomes of children with chronic disease globally: A systematic review. J. Paediatr. Child Health.

[20] Karatas C., Caldwell P.H., Scott K.M. (2022). How paediatricians communicate with parents who access online health information. J. Paediatr. Child Health.

[21] Janssen S., Fahlbusch F.B., Käsmann L., Rades D., Vordermark D. (2019). Radiotherapy for prostate cancer: DISCERN quality assessment of patient-oriented websites in 2018. BMC Urol..

[22] Janssen S., Käsmann L., Fahlbusch F.B., Rades D., Vordermark D. (2018). Side effects of radiotherapy in breast cancer patients: The Internet as an information source. Strahlenther. Onkol..

[23] Perzel S., Huebner H., Rascher W., Menendez-Castro C., Hartner A., Fahlbusch F.B. (2017). Searching the web: A survey on the quality of advice on postnatal sequelae of intrauterine growth restriction and the implication of developmental origins of health and disease. J. Dev. Orig. Health Dis..

[24] Nguyen S.K.A., Ingledew P.-A. (2013). Tangled in the Breast Cancer Web: An Evaluation of the Usage of Web-Based Information Resources by Breast Cancer Patients. J. Cancer Educ..

[25] Sacchetti P., Zvara P., Plante M.K. (1999). The internet and patient education—Resources and their reliability: Focus on a select urologic topic. Urology.

[26] Weissenberger C., Jonassen S., Beranek-Chiu J., Neumann M., Müller D., Bartelt S., Schulz S., Mönting J.S., Henne K., Gitsch G. (2004). Breast cancer: Patient information needs reflected in English and German web sites. Br. J. Cancer.

[27] Lee J.S. (2020). Digital communication, social media, and Englishes. World Englishes.

[28] Landis J.R., Koch G.G. (1977). The Measurement of Observer Agreement for Categorical Data. Biometrics.

[29] Charnock D., Shepperd S., Needham G., Gann R. (1999). DISCERN: An instrument for judging the quality of written consumer health information on treatment choices. J. Epidemiol. Commun. Health.

[30] Charnock D. (2004). Learning to DISCERN online: Applying an appraisal tool to health websites in a workshop setting. Health Educ. Res..

[31] Nghiem A.Z., Mahmoud Y., Som R. (2016). Evaluating the quality of internet information for breast cancer. Breast.

[32] Boyer C., Selby M., Scherrer J.R., Appel R.D. (1998). The Health On the Net Code of Conduct for medical and health Websites. Comput. Biol. Med..

[33] Silberg W.M. (1997). Assessing, Controlling, and Assuring the Quality of Medical Information on the Internet: Caveant Lector et Viewor—Let the Reader and Viewer Beware. JAMA.

[34] Hemmerich StatistikGuru: Cohen’s Kappa für zwei Rater Berechnen 2019. https://statistikguru.de/rechner/cohens-kappa-zwei-rater-berechnen.html.

[35] Altman D.G. (1990). Practical Statistics for Medical Research.

[36] Stemler S.E. A Comparison of Consensus, Consistency, and Measurement Approaches to Estimating Interrater Reliability. https://openpublishing.library.umass.edu/pare/article/id/1540/.

[37] Farouk Z.L., Usman F., Musa B.M., Ezeaka V.C., Okolo A. (2021). Societal awareness on neonatal hyperbilirubinemia: A systematic review and meta-analysis. Semin. Perinatol..

[38] Slusher T.M., Zamora T.G., Appiah D., Stanke J.U., Strand M.A., Lee B.W., Richardson S.B., Keating E.M., Siddappa A.M., Olusanya B.O. (2017). Burden of severe neonatal jaundice: A systematic review and meta-analysis. BMJ Paediatr. Open.

[39] Sgro M., Campbell D.M., Kandasamy S., Shah V. (2012). Incidence of Chronic Bilirubin Encephalopathy in Canada, 2007–2008. Pediatrics.

[40] Trotter A. (2021). Qualität der Versorgung sehr kleiner Frühgeborener in Deutschland—Auswertung öffentlich verfügbarer Daten der Perinatalzentren von 2014 bis 2018. Z. Geburtshilfe Neonatol..

[41] Lewandowski D., Schultheiß S. (2023). Public awareness and attitudes towards search engine optimization. Behav. Inf. Technol..

[42] Yilmaz A., Ozkiraz S., Akcan A.B., Canpolat M. (2015). Low-cost Home-use Light-emitting-diode Phototherapy as an alternative to Conventional Methods. J. Trop. Pediatr..

[43] Chang P.W., Waite W.M. (2020). Evaluation of Home Phototherapy for Neonatal Hyperbilirubinemia. J. Pediatr..

[44] Bevendorff J., Wiegmann M., Potthast M., Stein B., Goharian N., Tonellotto N., He Y., Lipani A., McDonald G., Macdonald C., Ounis I. (2024). Is Google Getting Worse? A Longitudinal Investigation of SEO Spam in Search Engines. Advances in Information Retrieval.

[45] Schaeffer D., Berens E.-M., Vogt D., Gille S., Griese L., Klinger J., Hurrelmann K. (2021). Health literacy in Germany. Dtsch. Ärztebl. Int..

[46] Schaeffer D., Berens E.-M., Vogt D. (2017). Health Literacy in the German Population. Dtsch. Ärztebl. Int..

[47] EF EPI (2023). EF English Proficiency Index A Ranking of 113 Countries and Regions by English Skills. https://www.ef.com/assetscdn/WIBIwq6RdJvcD9bc8RMd/cefcom-epi-site/reports/2023/ef-epi-2023-english.pdf.

[48] Statistisches Bundesamt (Destatis) Press Release No. 158 of 20 April 2023. https://www.destatis.de/EN/Press/2023/04/PE23_158_125.html.

[49] Berens E.-M., Klinger J., Mensing M., Carol S., Schaeffer D. (2022). Health Literacy of People with Migration Background in Germany: Results of the HLS-MIG (Short Summary).

