# Supplementary material for: Diving into the Digital Landscape: Assessing the Quality of Online Information on Neonatal Jaundice for Parents
Source: Children (Basel). 2024 Jul 19;11(7):877. doi: 10.3390/children11070877 (PMC11275572; doi:10.3390/children11070877)
Supplement: Supplementary file 1 [file children-11-00877-s001.zip › Supplementary Figure S1.pdf]

Diving into the Digital Landscape: Assessing the Quality of Online Information on Neonatal Jaundice for Parents. *Michael K. Baumgartner et al.*

Supplementary Figure S1

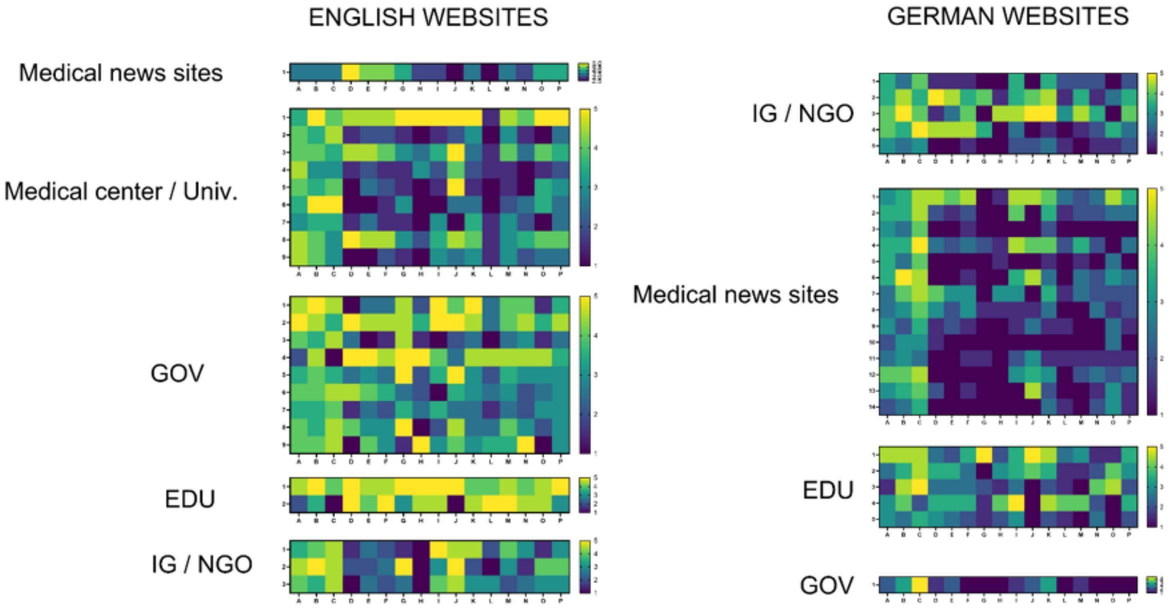

SF1. Heatmap analysis of the mean DISCERN Score in German and English websites on jaundice regarding the hosting source. Legend: y-axis 1 to 25 (German) and 1 to 24 (English) represents the top Google search hits, while the x-axis (A-P) represents DISCERN questions regarding reliability (A-H), treatment information (I-O) and the overall rating (P). Per item a minimum score of 1 (blue) and a maximum score of 5 (yellow) can be achieved. Med News = medical news site (sponsored), IG/NGO = interest groups / non-governmental organizations, EDU= educational, GOV = governmental, MedCenter/Univ = medical center / university.
