# Supplementary material for: Diving into the Digital Landscape: Assessing the Quality of Online Information on Neonatal Jaundice for Parents
Source: Children (Basel). 2024 Jul 19;11(7):877. doi: 10.3390/children11070877 (PMC11275572; doi:10.3390/children11070877)
Supplement: Supplementary file 1 [file children-11-00877-s001.zip › Supplementary File S1.pdf]

Standardized chart for the quality assessment of websites

Domain: ___________________________________

Google hit number (Top 20):_________________

**Category**<br>☐ Medical news site (sponsored)<br>☐ Medical centre/university<br>☐ Interest group/NGO<br>☐ Governmental<br>☐ Educational	
**Certification**<br>**HON code** (yes or no) (Boyer et al. 1998)<br>Other certification	☐ **Yes**<br>☐ **No**
**JAMA benchmarks** (yes or no) (Silberg et al. 1997)<br>☐ Authorship: *authors/contributors/affiliations*<br>☐ Attribution: *References/sources/copy right*<br>☐ Disclosure: *ownership/sponsors/support*<br>☐ Currency: *creation/update*	**Total score**<br>1 point for yes, each
**Modified DISCERN score** (Charnock et al. 1999)	
**Section 1 (1-5 points each)**	**Points**
1. **Are the aims clear?**<br>*Are the objectives of the website clearly defined? What is the subject matter? Which topics are addressed? Who is the target audience?*	
2. **Does it achieve its aims?**<br>*Does the website achieve its self-imposed goals?*	
3. **Is it relevant (readers` perspective)?**<br>*Questions that readers would ask, using understandable language?*	
4. **Sources of information**<br>*Are there clear indications of the information sources used in creating the publication (besides the author/producer)? How many sources are listed in the bibliography, references? Is the author clearly identified?*	
5. **Up-to-dateness of data**<br>*Is the creation date, updates, and dates of references clearly indicated? Is it distinctly stated when the information used and reproduced in the publication was created?*	
6. **Balanced against bias?**<br>*Is the presentation subjective? Are multiple sources cited, external review conducted? Is the publication written in a balanced and unbiased manner?*	
7. **Additional sources?**<br>*Does the publication contain detailed information about supplementary aids and resources?*	
8. **Refer to areas of uncertainty?**<br>Are different possibilities addressed? Is a possible lack of evidence mentioned? Does the publication address areas for which there is no secure information available? Is it indicated that only general advice is provided and should not be used for self-diagnosis or treatment?	

**Modified DISCERN score** (Charnock et al. 1999) -*continued*	
**Section 2: Modified questions (1-5 points each)**	**Points**
**9. Does the website describe the consequences or risks of jaundice?**	
**10. Does the website provide recommendations on when to contact a pediatrician?**	
**11. Does the website describe possible treatment options and the mechanism of action of each treatment method?**	
**12. Does the website describe the risks of each treatment procedure?**	
**13. Does the website describe the diagnostic procedure?**	
**14. Does the website describe how treatment procedures affect quality of life or the setting in which therapy is provided?**	
**15. Are recommendations made regarding pre- or post-treatment procedures?**	
**Section 3: Overall Rating of the Publication**	
**16. Finally, based on the answers to all of the preceding questions, evaluate the publication in terms of its overall quality as a source of information about jaundice-related consequences.**	
**Total modified DISCERN Score**	

Google search terms:

English language

1) “Jaundice newborn”
2) “Jaundice baby”

German language

3) “Neugeborenes Gelbsucht”
4) “Baby Gelbsucht”

Exclusion criteria

Limited information on search topic (<one paragraph)
Complete access restricted by password
Repeated server unavailability
Direct forwarding to other domains
